# Left Adrenal Gland Sampling Through Transesophageal Bronchoscopic Ultrasound-Guided Fine Needle Aspiration (EUS-B FNA): A New Route and a New Challenge

**DOI:** 10.7759/cureus.24571

**Published:** 2022-04-28

**Authors:** Parul, Monika Gupta, Pawan K Singh, Manjunath B Govindagoudar, Anindya Mittal

**Affiliations:** 1 Pulmonary and Critical Care Medicine, Pandit Bhagwat Dayal Sharma Post Graduate Institute of Medical Sciences (PGIMS), Rohtak, IND; 2 Pathology, Pandit Bhagwat Dayal Sharma Post Graduate Institute of Medical Sciences (PGIMS), Rohtak, IND; 3 Critical Care Medicine, Pandit Bhagwat Dayal Sharma Post Graduate Institute of Medical Sciences (PGIMS), Rohtak, IND

**Keywords:** adrenal metastasis, lung cancer, ebus, eus-b fna, left adrenal gland

## Abstract

Metastasis to adrenal glands from primary pulmonary carcinoma is quite a common occurrence. In most cases, the diagnosis is made based on an imaging evaluation done because of chronic non-specific pulmonary symptoms. Further evaluation to determine the type of carcinoma is done using histopathological evaluation of the primary lung lesion. Here, we have described a case of a 60-year-old man who presented with chest pain and was incidentally detected with a lower lung mass and a bulky left adrenal gland in the upper abdominal cuts on a CT of the thorax. As the evaluation of the fine needle aspiration (FNA) sample from lung lesion couldn't be successfully performed, sampling from the left adrenal gland was attempted under the guidance of conventional endoscopic ultrasound using an endobronchial ultrasound probe (EUS-B). When the technique failed to localise the left adrenal gland, a modification was made and the gland was localised using the spleen as a marker. This case further presented a challenge, when due to the unfolding of rugae, the FNA needle wasn’t able to reach up to the left adrenal gland. Further adjustment was made and the maximum depth of the gland from the margin was measured and the needle was fully freed. Multiple jabs were made and sampling was successfully done. Cellblock confirmed the presence of adenocarcinoma of pulmonary origin, positive for thyroid transcription factor 1 (TTF-1). The patient remained stable and did not present with any early or late post-procedural complications. The patient was started on appropriate chemotherapy for the disease. He has received three cycles of carboplatin and pemetrexed till now and is doing well.

## Introduction

Lung cancer is one of the leading causes of cancer mortality [1]. The diagnosis of lung cancer is based on imaging modalities and histopathological examination of the lesion. Adrenal glands are one of the most common sites of metastasis from lung cancer [2] and therefore the European Guidelines recommend endosonography for tissue verification from left adrenal gland lesions suspected of metastases [3]. Sampling the left adrenal using endoscopic ultrasound-guided fine needle aspiration (EUS-FNA) for the staging of lung cancer is a common practice and its use for primary diagnosis of lung cancer has been previously described in the literature [4]. With the advancement in imaging techniques, endoscopic ultrasound-guided fine needle aspiration (EUS-FNA) is now being extensively used for visualization and tissue sampling from the left adrenal gland. More recently, the use of endo-bronchial ultrasound (EBUS) probe to perform both bronchoscopy and EUS-FNA of the left adrenal gland in the same setting has been described [3,5,6]. Even though a conventional technique has been defined extensively in the literature [5], on facing difficulty, the technique has to be modified according to case requirements. Here we have discussed one such case in which the conventional technique failed to visualize the left adrenal gland and so certain modifications had to be made to establish the primary diagnosis of lung adenocarcinoma.

## Case presentation

**Figure 1:**
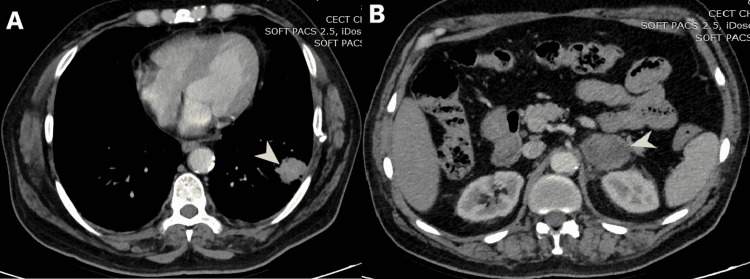
(A) CT scan of thorax, (B) Upper abdomen section of the same scan A: Mediastinal window of contrast-enhanced CT of the thorax showing 3 cm x 2.5 cm heterogeneously enhancing mass-like lesion (arrowhead) in the lower lobe of left lung reaching up to the pleura. B: Upper abdomen sections of the same scan show bulky heterogeneously enhancing left adrenal gland (arrowhead) measuring 6 cm x 4.5 cm.

A 60-year-old-man presented with chest pain and incidentally detected left lower lobe lung mass (Figure 1, A) and a bulky left adrenal gland (LAG) (Figure 1, B) in upper-abdomen cuts on CT of the thorax. A PET-CT of the patient showed increased uptake in the left lower lobe of the lung and left adrenal gland. 

**Figure 2:**
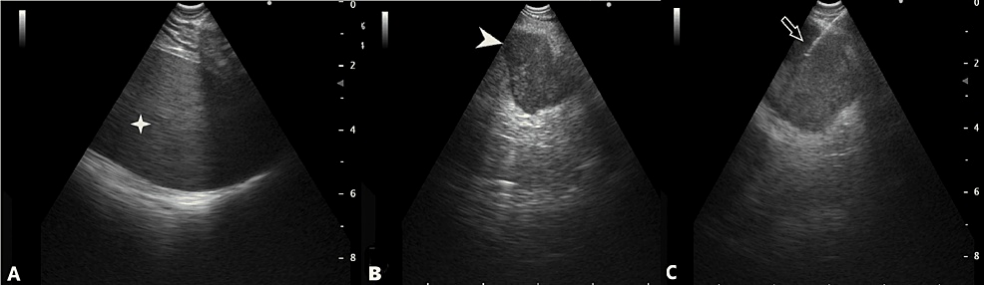
Endobronchial ultrasound images with the transducer placed in the stomach showing the spleen A: The sliding counter-clockwise and caudally left adrenal gland is visible (indicated by the star), B: Bulky adrenal is visible (marked by the arrowhead), C: Needle (marked by the arrow) placed inside the adrenal gland

Ultrasound-guided followed by CT-guided FNA from lung lesion was attempted. Bronchoscopy was done along with radial-endobronchial-ultrasound scanning, but the lesion couldn’t be localized. In the same sitting, it was planned that FNA will be attempted from the left adrenal gland with conventional-endobronchial-ultrasound-guidance (cEBUS) as has been described previously [6]. Under conscious sedation, cEBUS was passed through the esophagus. As per the conventional approach, the left adrenal gland couldn’t be localized, and a new technique was tried. From the anteriorly-placed left lobe of the liver, EBUS was turned left to look for the spleen (visible as a homogenous organ in Figure 2, A). Further, the transducer was turned posteriorly (further counterclockwise) and caudally, where bulky adrenal was visible (Figure 2, B).

**Figure 3:**
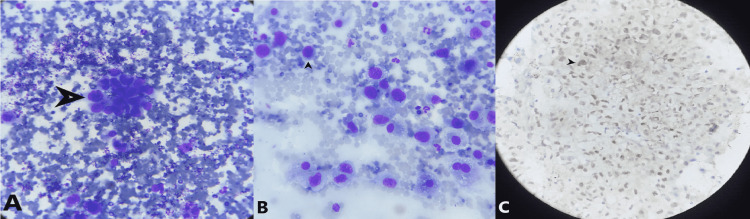
Fine needle aspiration cytology examination of sample from the left adrenal gland A: Cluster of tumor cells under 100x magnification, B: Individual cells showing high nucleocytoplasmic ratio with pleomorphic nuclei and prominent nucleoli, C: Immunocytochemistry showing nuclear positivity of thyroid transcription factor-1 (TTF1)

The transducer was no longer near the gastro-oesophageal junction and had slipped into the stomach [7]. Major vascularity was ruled out and FNA using a 21G-needle with a 3 cm length lock was attempted under guidance. Despite jabs under guidance, due to the unfolding of rugae, the needle tip was not able to reach up to the left adrenal gland. Hence, another adjustment was made, the maximum depth of the left adrenal gland from the margin was measured (7.4 cm) and the needle was fully freed (5 cm). The following attempt was brisk and swift. This sudden move was able to place the tip of the needle into the center of the lesion. Multiple jabs were made, samples were taken for slides, and formalin preserved cell block. The rapid on-site evaluation confirmed the presence of adenocarcinoma. There were no post-procedure complications. An ultrasound of the abdomen done six hours later did not reveal any free-fluid. Cellblock confirmed the presence of adenocarcinoma with pulmonary origin (TTF-1 positive) (Figure 3).

Molecular aberrations were negative, and the patient was started on chemotherapy. The patient has since received three cycles of carboplatin and pemetrexed and is doing well.

## Discussion

Lung cancer is one of the leading causes of cancer-related mortality [1]. Non-specific presentation often causes a delay in the diagnosis. Nevertheless, irrespective of the time or stage of presentation, diagnosis of lung cancer requires a combination of imaging modalities and histopathological examination to guide further treatment. The majority of lung cancer cases are non-small cell lung cancer variants (NSCLC). Adrenal glands are one of the most common sites for metastasis from lung cancer [2]. While 2/3rd of these masses are benign [8], the possibility of metastasis warrants the need for biopsy. The European Guidelines recommend endosonography for tissue verification from LAG lesions suspected of metastases [3].

With the advancements in imaging and biopsy techniques, EUS-FNA has been extensively described in the literature for effective visualization and sampling of adrenal glands. A EUS-FNA can be performed either with the use of a conventional gastrointestinal ultrasound scope (EUS) or by using the EBUS-scope in the esophagus (EUS-B) [3]. Biopsy of the adrenal gland by EUS-B- FNA has shown sample adequacy of 87%[8], which is comparable to that of conventional EUS. Left adrenal gland analysis by EUS-B shows a similar high success rate as conventional EUS [6] . The advantage of EUS-B is that complete mediastinal, hilar and left adrenal gland staging can be performed in a single endoscopy procedure using just an EBUS scope [6]. As per conventional technique, to identify the left adrenal gland, the tip of the bronchoscope is slid down the aorta up to the celiac trunk and mesenteric artery, which are the first vessels extending from the abdominal aorta. At this point, the EUS-B scope is turned counterclockwise (if the physician is standing behind a patient lying in a supine position this movement turns the transducer to the left) to visualize the left kidney, the spleen, and the left adrenal gland just behind the celiac trunk and mesenteric artery [5]. While the technique has high effectiveness, it doesn’t guarantee a 100% success rate and so, as per the requirement of the case, it had to be modified when it failed to localize the left adrenal gland. The posteriorly located spleen was instead used as a marker to localize the left adrenal gland. By doing this modification in the procedure, the left adrenal gland was identified successfully.

Furthermore, in this case, the difficulty was faced while attempting FNA in a conventional way due to the unfolding of rugae, as the needle tip was not able to reach the left adrenal gland. Hence, another adjustment was made, the maximum depth of the left adrenal gland from the margin was measured (7.4 cm) and the needle was fully freed (up to 5 cm). The following attempt was swift and brisk. This sudden move was able to place the tip of the needle into the center of the lesion, piercing the gastric wall without unfolding the rouge. Multiple jabs were made, samples were taken for slides, and formalin-preserved cell block.

In the past, adrenal mass sampling has been done for the staging of lung cancer. The use of adrenal gland sampling by EUS-FNA for the primary diagnosis of lung cancer has been previously described in the literature [4]. In the current study, as the biopsy from the lung mass couldn't be performed successfully, the sampling from the enlarged left adrenal gland by EUS-B FNA was used to make a definitive diagnosis and further treatment guidance. The lung mass found on the CT scan, and the increased uptake on PET-CT and TTF-1 positivity of the biopsy sample obtained from the left adrenal gland, confirmed adenocarcinoma of pulmonary origin thereby aiding in reaching a definitive diagnosis. Here we have described a technique for left adrenal gland localization and sampling. We used left adrenal gland sampling for the primary diagnosis of the patient, and not just staging [8,9].

## Conclusions

In case the difficulty is encountered in localizing the adrenal gland using conventional EUS-B, the technique can be modified according to the case requirement. From the anteriorly placed left lobe of the liver, the transducer can be turned to the left to look for the spleen which can be used as a marker to localize the left adrenal gland which can be found further posteriorly and caudally. Furthermore, on facing difficulty with FNA sampling due to the unfolding of rugae, the distance from the margin to the left adrenal gland can be calculated and the needle can be freed. The needle can then be placed in the center of the lesion with a swift and brisk movement. Histopathological examination of the FNA sample from the adrenal gland combined with imaging modalities can be used to make the diagnosis of primary lung carcinoma in case the histopathological examination of lung lesion turns out to be inconclusive.

## References

[1] Siegel RL, Miller KD, Fuchs HE, Jemal A (2022). Cancer statistics, 2022. CA Cancer J Clin.

[2] Milovanovic IS, Stjepanovic M, Mitrovic D (2017). Distribution patterns of the metastases of the lung carcinoma in relation to histological type of the primary tumor: an autopsy study. Ann Thorac Med.

[3] Vilmann P, Frost Clementsen P, Colella S (2015). Combined endobronchial and esophageal endosonography for the diagnosis and staging of lung cancer: European Society of Gastrointestinal Endoscopy (ESGE) Guideline, in cooperation with the European Respiratory Society (ERS) and the European Society of Thoracic Surgeons (ESTS). Eur J Cardiothorac Surg.

[4] Liu M, Zhang Q, Long H, Xu M, Shou Y, Guo Z (2018). Diagnosis of lung adenocarcinoma with left adrenal metastasis via transesophageal endoscopic ultrasound-guided fine-needle aspiration biopsy: a case report. Mol Clin Oncol.

[5] Bugalho A, de Santis M, Slubowski A, Rozman A, Eberhardt R (2017). Trans-esophageal endobronchial ultrasound-guided needle aspiration (EUS-B-NA): a road map for the chest physician. Pulmonology.

[6] Crombag LM, Szlubowski A, Stigt JA, Schuurbiers O, Korevaar DA, Bonta PI, Annema JT (2017). EUS-B-FNA vs conventional EUS-FNA for left adrenal gland analysis in lung cancer patients. Lung Cancer.

[7] Eloubeidi MA (2008). Endoscopic ultrasound in the evaluation of adrenal masses. Gastroenterol Hepatol (N Y).

[8] Christiansen IS, Ahmad K, Bodtger U (2020). EUS-B for suspected left adrenal metastasis in lung cancer. J Thorac Dis.

[9] Meena N, Hulett C, Patolia S, Bartter T (2016). Exploration under the dome: asophageal ultrasound with the ultrasound bronchoscope is indispensible. Endosc Ultrasound.

